# Identification of Palliative Care Needs and Mental Health Outcomes Among Family Members of Patients With Severe Acute Brain Injury

**DOI:** 10.1001/jamanetworkopen.2023.9949

**Published:** 2023-04-25

**Authors:** Wesley V. Plinke, Stephanie A. Buchbinder, Lyndia C. Brumback, W. T. Longstreth, Whitney A. Kiker, Robert G. Holloway, Ruth A. Engelberg, J. Randall Curtis, Claire J. Creutzfeldt

**Affiliations:** 1 Department of Medicine, University of Washington, Seattle; 2 Department of Epidemiology, University of Washington, Seattle; 3 Department of Biostatistics, University of Washington, Seattle; 4 Department of Neurology, University of Washington, Seattle; 5 Division of Pulmonary, Critical Care and Sleep Medicine, University of Washington, Seattle; 6 Department of Neurology, University of Rochester, Rochester, New York; 7 Cambia Palliative Care Center of Excellence at UW Medicine, University of Washington, Seattle

## Abstract

**Importance:**

Family members of patients with severe acute brain injury (SABI) are at risk for poor psychological outcomes.

**Objective:**

To explore the utility of the early use of a palliative care needs checklist in identifying care needs of patients with SABI and family members who are at risk of poor psychological outcomes.

**Design, Setting, and Participants:**

This prospective cohort study included patients with SABI in an intensive care unit (ICU) for 2 days or more and a Glasgow Coma Scale score of 12 or lower and their family members. This single-center study was conducted at an academic hospital in Seattle, Washington, from January 2018 to June 2021. Data were analyzed from July 2021 to July 2022.

**Exposure:**

At enrollment, a 4-item palliative care needs checklist was completed separately by clinicians and family members.

**Main Outcomes and Measures:**

A single family member for each enrolled patient completed questionnaires assessing symptoms of depression and anxiety, perception of goal-concordant care, and satisfaction in the ICU. Six months later, family members assessed their psychological symptoms, decisional regret, patient functional outcome, and patient quality of life (QOL).

**Results:**

A total of 209 patient–family member pairs (family member mean [SD] age, 51 [16] years; 133 women [64%]; 18 Asian [9%], 21 Black [10%], 20 [10%] Hispanic, and 153 White [73%] participants) were included. Patients had experienced stroke (126 [60%]), traumatic brain injury (62 [30%]), and hypoxic-ischemic encephalopathy (21 [10%]). At least 1 need was identified for 185 patients or their families (88%) by family members and 110 (53%) by clinicians (κ = −0.007; 52% agreement). Symptoms of at least moderate anxiety or depression were present in 50% of family members at enrollment (87 with anxiety and 94 with depression) and 20% at follow-up (33 with anxiety and 29 with depression). After adjustment for patient age, diagnosis, and disease severity and family race and ethnicity, clinician identification of any need was associated with greater goal discordance (203 participants; relative risk = 1.7 [95% CI, 1.2 to 2.5]) and family decisional regret (144 participants; difference in means, 17 [95% CI, 5 to 29] points). Family member identification of any need was associated with greater symptoms of depression at follow-up (150 participants; difference in means of Patient Health Questionnaire–2, 0.8 [95% CI, 0.2 to 1.3] points) and worse perceived patient QOL (78 participants; difference in means, −17.1 [95% CI, −33.6 to −0.5] points).

**Conclusions and Relevance:**

In this prospective cohort study of patients with SABI and their families, palliative care needs were common, although agreement on needs was poor between clinicians and family members. A palliative care needs checklist completed by clinicians and family members may improve communication and promote timely, targeted management of needs.

## Key Points

**Question:**

For patients with severe acute brain injury (SABI) and their families, is there an association between palliative care needs identified on a survey and mental health outcomes in family members?

**Findings:**

This cohort study of 209 patient–family member dyads found that palliative care needs after SABI were common, although clinicians and family members identified different needs. Psychological distress at follow-up was also common and associated with early identification of palliative care needs by family members.

**Meaning:**

The results of this study suggest that patients with SABI and their family members have many palliative care needs, and improved communication between clinicians and family members about these needs may help address them and improve mental health outcomes.

## Teaser

This cohort study explores the agreement between clinicians and family members of patients with severe acute brain injury regarding patient palliative care needs and assesses the association of identified needs with family member psychological outcome.

## Introduction

Family members of patients in the intensive care unit (ICU) are at increased risk for poor psychological outcomes.^1,2^ High levels of stress and feelings of uncertainty associated with the patient’s prognosis, treatment, and goals of care^3^ can lead to poor outcomes for family members, including symptoms of anxiety and depression^4,5,6,7^ during the ICU stay and after discharge.^1,8,9^ Long-term psychological distress may be particularly prevalent in family members of patients with severe acute brain injury (SABI).^10^

Palliative care offers a strategy to identify and address the needs of critically ill patients and their family members.^11,12^ Randomized clinical trials using palliative care interventions to improve patient and family member outcomes^13,14,15^ largely enrolled patients based on illness type or severity, yet experts have highlighted the importance of targeting interventions based on palliative care needs.^16,17^ We hypothesized that early identification of palliative care needs of patients with SABI and their family members would identify those at highest risk for poor outcomes and may aid in the design of timely and targeted interventions to improve outcomes. Our research questions were (1) to what extent do family members and clinicians agree on the presence of palliative care needs early in the ICU stay and (2) are identified needs associated with family member psychological outcome, including symptoms of depression, anxiety, or decisional regret, and with patient outcomes of family-reported goal concordant care and perceived patient quality of life (QOL).

## Methods

We developed a palliative care needs checklist through literature review, local focus groups, and expert opinion and refined an initial version^18^ based on further stakeholder input. The revised SuPPOrTT checklist (Figure) consists of 4 questions around support needs, pain or other symptoms, prognosis and treatment options, and treatment targets (goals of care). The SuPPOrTT study was a prospective cohort study conducted from January 2018 to June 2020 at an academic county hospital with a comprehensive stroke and level I trauma center in Seattle, Washington. The checklist was completed by clinicians on ICU work rounds and by family members on the day of enrollment into the study.

**Figure.**
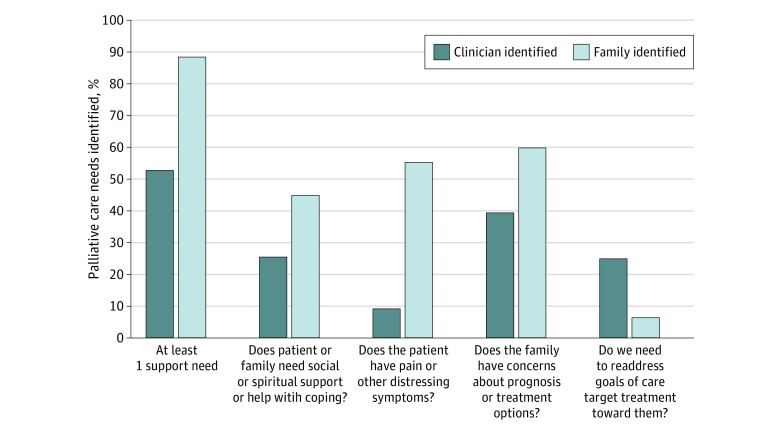
Palliative Care Needs Identified by Clinicians and Family Members Palliative care needs were identified using the SuPPOrTT checklist as indicated. Results are presented as percentages. The κ statistic reflects agreement between family and clinician identified needs, as follows: at least 1 support needed, κ = −0.007 (95% CI, −0.097 to 0.082; 51.7% agreement); social or spiritual support, κ = −0.01 (95% CI, −0.14 to 0.11; 52.2% agreement); pain or other distressing symptoms, κ = 0.06 (95% CI, −0.009 to 0.13; 48.3% agreement); prognosis or treatment, κ = 0.01 (95% CI, −0.11 to 0.13; 48.3% agreement); and goals of care, κ = 0.07 (95% CI, −0.05 to 0.18; 74.2% agreement).

### Participants and Enrollment

We identified consecutive patients admitted to ICUs with a diagnosis of SABI, defined as stroke (ischemic stroke, intraparenchymal hemorrhage, or subarachnoid hemorrhage), traumatic brain injury, or hypoxic-ischemic encephalopathy. Patients with SABI were eligible between ICU day 2 and 14 if they were 18 years or older, had a Glasgow Coma Scale (GCS) score of 12 or less, had an English-speaking family member available at bedside or by telephone, and lacked an established decision to withdraw life-sustaining treatment. Family members were approached after receiving permission from the medical team. If multiple family members were interested, the one that took chief responsibility for decision-making was asked to complete the survey after providing consent. Given that patients were unable to consent themselves, the legal surrogate decision-maker was asked to consent to review of patients’ electronic health record (EHR). Patients were included in the study only if both the clinicians and families had completed the palliative care needs checklist. The University of Washington institutional review board approved this study, and we followed the Strengthening and Reporting of Observational Studies in Epidemiology (STROBE) reporting guideline. The legal surrogate decision-maker provided written informed consent.

### Variables

The primary variables of interest included the identification of any palliative care need (out of 4) by clinicians and separately by families. Additional variables of interest included identification of each of the 4 needs. Clinician-identified palliative care needs were defined by the SuPPOrTT checklist that was completed daily by clinicians on morning work rounds. We collected those completed on the day of enrollment. On the same day, research staff asked family members the questions from the SuPPOrTT checklist, and those were used to define family-identified palliative care needs.

### Outcomes

The family members who completed the survey at enrollment again completed a survey at follow up approximately 6 months later (eTable 1 in Supplement 1). Our main outcome was psychological distress in family members at follow-up. We administered the Patient Health Questionnaire–2 (PHQ-2)^19^ and the General Anxiety Disorder–7 (GAD-7)^20^ at enrollment and follow-up. The PHQ-2 is scored from 0 to 6 with a score of 3 or higher suggesting a high level of depressive symptoms. The GAD-7 is scored from 0 to 21 with scores 10 or higher suggesting moderate anxiety or worse. The goal was not to diagnose depression or anxiety but to examine the degree of psychological distress as identified by high levels of depressive or anxiety symptoms. Additional outcomes included family satisfaction with care while in the ICU assessed at enrollment and decisional regret in family members at follow-up. Family satisfaction was assessed using the FS-ICU survey,^21^ a validated 24-item tool that measures satisfaction with care and decision-making with scores ranging from 0 to 100, with higher scores indicating greater satisfaction. Decisional regret was assessed using the decision regret scale (DRS),^22^ a 5-item scale that asks participants to reflect on a particular decision they made and rate each item on a scale from 1 (strongly agree) to 5 (strongly disagree). Total DRS scores range from 0 to 100, and higher scores indicate greater regret. When responses to individual survey questions were missing, we followed the instructions of the survey developers to impute the missing response.^19,20,21,22^

Anticipating that most patients would be unable to participate in surveys, even at follow-up, we assessed patient outcomes from the perspective of the family member, including perception of goal-discordant care in the enrollment survey and assessment of patient functional recovery and patient QOL at follow-up. To assess goal-discordant care, we used 2 questions that were adapted to SABI from the landmark SUPPORT study.^3,23^ We asked family members, “If your loved one were able and had to make a choice today, would they prefer a plan of medical care that focuses on extending life as much as possible, or would they want a plan of medical care that focuses on comfort and would limit life-saving treatment?” Possible responses were, “All efforts to prolong life as much as possible,” “Limit life-saving treatment and focus on comfort,” and “I’m not sure which they would choose.” We then asked the family member, “Which of the following best describes the focus of the medical care your loved one is currently receiving?” with the same possible answers as before. Goal-discordant care was determined when the answers to these 2 questions were discordant, across the 3 answers including “I’m not sure.”^3^ For family assessment of patient functional recovery at follow-up, we used the modified Rankin Scale (mRS) score. If no follow-up survey was completed, we were able to abstract patient 6-month mRS from return clinic visits using the EHR.^24^ To ensure that mRS determined by family members and EHR review were consistent, we compared family-reported mRS with mRS by EHR review for 15% of patients and found no discrepancies. Perceived patient QOL was measured using the Euro-QoL visual analog scale, whereby higher scores on a scale from 0 to 100 reflect better QOL. Length of stay in the ICU in days was abstracted from the EHR.

### Covariates

The survey also included self-reported information on sociodemographic characteristics of the family member (age, gender, race, ethnicity, level of education, relationship to the patient). We collected patient sociodemographic and clinical data including age, gender, race, ethnicity, and primary diagnosis from the EHR. Race and ethnicity were dichotomized into (1) White and (2) race other than White or Hispanic, which included Alaska Native, American Indian, Asian, Black or African American, Hispanic ethnicity, Pacific Islander, or another unspecified race.

### Statistical Analysis

To assess agreement between family and clinician identification of palliative care needs (≥1 need and for each of the 4 needs individually), we calculated Cohen κ statistic and percentage agreement, along with the percentage of families and clinicians who identified needs. To evaluate the association between palliative care needs and outcomes in this exploratory study, we used linear regression or a Poisson working model (for the binary outcomes goal-discordant care and death within 6 months) with robust standard errors.

We fit unadjusted models and models adjusted for patient age, diagnosis and disease severity (GCS for outcomes at enrollment and mRS for outcomes at 6 months) and family race and ethnicity. For each outcome, we included all participants without missing data for that outcome and covariates; this allowed more participants to be included in the analysis for each outcome but assumed that the different samples were similar (for comparisons across outcomes) and representative of the target population.

Statistical analyses were conducted using R version 4.0.2 (R Foundation for Statistical Computing). We used *P* < .05 to signify statistical significance for all analyses, and all tests were 2-tailed.

## Results

Among the 222 enrolled patient and family member pairs, both clinician and family SuPPOrTT checklists were completed by 209 (94%). Patient mean (SD) age was 58 (19) years, 94 (45%) were women, and 21 (10%) were Asian, 20 (10%) were Black, 14 (7%) were Hispanic, and 158 (76%) were White participants (Table 1). Family member mean (SD) age was 51 (16) years, 133 (64%) were women, and 18 (9%) were Asian, 21 (10%) were Black, 20 (10%) were Hispanic, and 153 (73%) were White participants. The most common relationships of the family member to the patient were adult child (73 [35%]) and spouse (67 [32%]). Patients were most often hospitalized for stroke (126 [60%]), with 62 (30%) with traumatic brain injury and 21 (10%) with hypoxic-ischemic encephalopathy. At the time of enrollment (median [IQR] hospital day, 4 [3-6]), mean (SD) GCS for patients was 7.2 (2.6). More than one-third of enrolled patients died in the hospital (82 [37%]).

**Table 1.** Selected Demographic Characteristics of the Study Sample

Characteristic	Participants, No. (%)	
	Patients (N = 209)	Family members (N = 209)
Age, mean (SD), y	58.00 (18.74)	51.14 (15.88)
Gender		
Women	94 (44.98)	133 (63.64)
Men	115 (55.02)	76 (36.36)
Race		
Asian	21 (10.05)	18 (8.61)
Black	20 (9.57)	21 (10.05)
American Indian, Alaska Native, or Pacific Islander	10 (4.78)	12 (5.74)
Other/did not answer^a^	NA	5 (2.39)
White	158 (75.60)	153 (73.21)
Hispanic ethnicity	14 (6.7)	20 (9.61)
Race and ethnicity other than White and non-Hispanic^b^	65 (31.1)	70 (33.5)
Diagnosis		
Stroke	126 (60.29)	NA
Traumatic brain injury	62 (29.67)	NA
Hypoxic-ischemic encephalopathy	21 (10.05)	NA
GCS at enrollment, mean (SD)	7.24 (2.62)	NA
Relationship to patient		
Spouse	NA	67 (32.06)
Child	NA	73 (34.93)
Other	NA	69 (33.01)

Abbreviations: GCS, Glasgow Coma Scale; NA, not applicable.

^a^ Includes any race other than those specified.

^b^ Includes Alaska Native, American Indian, Asian, Black or African American, Hispanic ethnicity, Pacific Islander, or another unspecified race.

Among the 188 family members who responded to questions about anxiety and depressive symptoms at enrollment, the mean (SD) PHQ-2 was 2.5 (1.9), and 94 (50%) had a high level of depressive symptoms (PHQ-2 ≥3). The mean (SD) GAD-7 was 9.2 (5.9), and 87 (46%) had at least moderate anxiety (GAD-7 ≥10).

The follow-up survey was returned by family members of 155 of 209 patients (74%) at a mean (SD) of 146.3 (48.8) days (4.8 months) after enrollment. Functional outcome by follow-up was unknown for 11 of 209 patients (5.0%); 92 of 198 (46.5%) had died (mRS score, 6); and 106 survivors had a mean (SD) mRS of 3.5 (1.2). At follow-up, 150 family members provided information about depressive symptoms and 147 about anxiety. In these respective groups, the mean (SD) PHQ-2 was 1.7 (1.7), and 29 (19%) had a high level of depressive symptoms, and the mean (SD) GAD-7 was 6.2 (4.8), and 33 (22%) had at least moderate anxiety (Table 2).

**Table 2.** Association of 1 or More Needs With Outcomes^a^

Outcome	Mean (SD)	Clinician identification		Family-member identification	
		β (95% CI)^b^	*P* value	β (95% CI)^b^	*P* value
PHQ-2 at enrollment (n = 188)	2.53 (1.94)	0.42 (−0.20 to 1.04)	.19	0.12 (−0.84 to 1.08)	.80
PHQ-2 at follow-up (n = 150)	1.68 (1.68)	−0.40 (−0.93 to 0.14)	.14	0.75 (0.24 to 1.25)	.004
GAD-7 at enrollment (n = 188)	9.21 (5.93)	1.35 (−0.55 to 3.24)	.16	1.87 (−0.44 to 4.18)	.11
GAD-7 at follow-up (n = 147)	6.21 (4.79)	−0.30 (−1.96 to 1.36)	.72	1.56 (−0.51 to 3.63)	.14
Goal discordance (n = 203), No. (%)	82 (40)	1.74 (1.20 to 2.53)^c^	.004	0.79 (0.53 to 1.20)^c^	.27
FS-ICU at enrollment (n = 187)	89.3 (11.3)	−0.52 (−4.42 to 3.37)	.79	−2.15 (−5.16 to 0.87)	.16
ICU length of stay, d (n = 209)	15.9 (11.7)	1.65 (−1.81 to 5.12)	.35	−2.14 (−7.49 to 3.21)	.43
mRS at follow-up (n = 198)	4.64 (1.55)	0.07 (−0.39 to 0.53)	.76	−0.17 (−0.85 to 0.50)	.61
Death at follow-up (n = 198), No. (%)	92 (47)	1.11 (0.80 to 1.53)^c^	.54	0.90 (0.59 to 1.36)^c^	.62
QOL at follow-up (n = 78)	51.4 (23.2)	−2.72 (−13.80 to 8.36)	.63	−17.08 (−33.64 to −0.52)	.04
Decisional regret at follow-up (n = 144)	17.2 (21.4)	17.33 (5.42 to 29.25)	.005	−1.62 (−16.99 to 13.74)	.83

Abbreviations: FS-ICU, Family Satisfaction in the Intensive Care Unit; GAD-7; Generalized Anxiety Disorder–7; ICU, intensive care unit; mRS, modified Rankin Scale; PHQ-2, Patient Health Questionnaire–2; QOL, quality of life.

^a^ Fully adjusted models were adjusted for patient age, diagnosis, disease severity (using Glasgow Coma Scale at baseline and mRS at follow-up) and family self-reported race and ethnicity.

^b^ Linear regression models with robust standard errors were fit for continuous outcomes, and Poisson regression models with robust standard errors were fit for dichotomous outcomes of goal discordance at enrollment and death at follow-up.

^c^ Values are relative risks with 95% CIs.

### Identification of at Least 1 Need by Clinicians and Family Members

Using the SuPPOrTT checklist for 209 patients, clinicians identified at least 1 palliative care need for 110 patients (52.6%), while family members identified at least 1 for 185 patients (88.2%) (κ = −0.007 [95% CI, −0.097 to 0.082]; 52% agreement) (Figure). Clinicians identified a need to readdress goals of care more often than family members. There was poor agreement about any need or each of the 4 needs between clinicians and family members, with κ statistics ranging from −0.012 to 0.069 and percentage agreement ranging from 48% to 74% (Figure).

### Association of Clinician-Identified Palliative Care Needs With Outcomes

In the adjusted models (Table 2), we did not find an association between the main outcomes of anxiety and depressive symptoms and at least 1 clinician-identified need. Compared with no identification of need, identification of at least 1 need by clinicians was associated with a 74% higher proportion of perceived goal-discordant care at enrollment (relative risk [RR], 1.74; 95% CI, 1.20-2.53; *P* = .004) and with 17 points greater decisional regret, on average, at follow-up (difference in means, 17.33 points; 95% CI, 5.42-29.25 points; *P* = .005), among families of the same race and ethnicity and whose patients were the same age and had the same diagnosis and disease severity. eTable 2 in Supplement 1 shows results from the unadjusted models.

Among individual clinician-identified needs (Table 3), social and/or spiritual support or help with coping was significantly associated with higher family depressive symptoms at enrollment (difference in means for PHQ-2, 0.87 points; 95% CI, 0.10-1.63 points; *P* = .03). Clinician-identified presence of pain or other distressing symptoms for the patient was significantly associated with higher family symptoms of anxiety at follow-up (difference in means for GAD-7, 3.60 points, 95% CI, 0.18-7.03 points; *P* = .04) and with higher decisional regret (difference in means for DRS, 22.77 points; 95% CI, 3.27-42.28 points; *P* = .02). The individual clinician-identified need of a concern about prognosis or treatment options was also significantly associated with higher decisional regret (difference in means for DRS, 17.58 points; 95% CI, 4.14-31.03 points; *P* = .01).

**Table 3.** Association of Individual Needs With Outcomes^a^

Outcome	Clinician identification		Family-member identification	
	β (95% CI)^b^	*P* value	β (95% CI)^b^	*P* value
**Need for social or spiritual support, help with coping**				
PHQ-2 at enrollment (n = 188)	0.87 (0.10 to 1.63)	.03	0.55 (−0.01 to 1.10)	.05
PHQ-2 at follow-up (n = 150)	0.49 (−0.17 to 1.15)	.14	0.34 (−0.19 to 0.88)	.21
GAD-7 at enrollment (n = 188)	2.13 (−0.18 to 4.46)	.07	1.20 (−0.48 to 2.89)	.16
GAD-7 at follow-up (n = 147)	−0.20 (−2.09 to 1.69)	.83	0.82 (−0.72 to 2.36)	.30
Goal discordance (n = 203)	0.98 (0.62 to 1.55)^c^	.93	0.96 (0.68 to 1.34)^c^	.79
Decisional regret at follow-up (n = 144)	4.84 (−3.38 to 13.07)	.24	−1.24 (−8.19 to 5.70)	.72
**Patient pain or distressing symptoms**				
PHQ-2 at enrollment (n = 188)	−0.31 (−1.43 to 0.80)	.58	0.32 (−0.23 to 0.87)	.26
PHQ-2 at follow-up (n = 150)	0.20 (−0.74 to 1.13)	.68	0.04 (−0.50 to 0.58)	.89
GAD-7 at enrollment (n = 188)	−0.08 (−3.74 to 3.59)	.97	1.32 (−0.44 to 3.08)	.14
GAD-7 at follow-up (n = 147)	3.60 (0.18 to 7.03)	.04	1.60 (0.02 to 3.19)	.047
Goal discordance (n = 203)	1.52 (0.91 to 2.56)^c^	.11	0.85 (0.60 to 1.19)^c^	.34
Decisional regret at follow-up (n = 144)	22.77 (3.27 to 42.28)	.02	2.97 (−4.08 to 10.01)	.41
**Concern about prognosis or treatment options**				
PHQ-2 at enrollment (n = 188)	0.35 (−0.33 to 1.03)	.31	−0.38 (−0.96 to 0.19)	.19
PHQ-2 at follow-up (n = 150)	−0.04 (−0.6 to 0.54)	.89	−0.35 (−0.91 to 0.22)	.22
GAD-7 at enrollment (n = 188)	1.61 (−0.48 to 3.70)	.13	0.33 (−1.40 to 2.07)	.70
GAD-7 at follow-up (n = 147)	0.39 (−1.40 to 2.18)	.67	−0.34 (−1.91 to 1.24)	.67
Goal discordance (n = 203)	1.29 (0.88 to 1.89)^c^	.19	0.95 (0.67-1.22)^c^	.75
Decisional regret at follow-up (n = 144)	17.58 (4.14 to 31.03)	.01	−15.81 (−34.32 to 2.69)	.09
**Need to readdress or change goals of care**				
PHQ-2 at enrollment (n = 188)	0.39 (−0.34 to 1.12)	.30	0.51 (−0.66-1.68)	.39
PHQ-2 at follow-up (n = 150)	0.51 (−0.22 to 1.24)	.17	−0.75 (−1.72 to 0.22)	.13
GAD-7 at enrollment (n = 188)	1.92 (−0.49 to 4.33)	.12	0.74 (−3.19 to 4.67)	.71
GAD-7 at follow-up (n = 147)	−0.00 (−2.25 to 2.24)	>.99	−3.25 (−4.62 to −1.88)	<.001
Goal discordance (n = 203)	1.36 (0.93 to 1.98)^c^	.12	1.59 (1.03 to 2.44)^c^	.04
Decisional regret at follow-up (n = 144)	13.82 (−0.29 to 27.94)	.05	17.36 (−9.05 to 43.78)	.20

Abbreviations: GAD-7, Generalized Anxiety Disorder–7; PHQ-2, Patient Health Questionnaire–2.

^a^ Fully adjusted models were adjusted for patient age, diagnosis, disease severity (using either Glasgow Coma Scale or modified Rankin Scale) and family self-reported race/ethnicity.

^b^ Linear regression models with robust standard errors were fit for continuous outcomes, and Poisson regression models with robust standard errors were fit for dichotomous outcomes of goal discordance at enrollment and death at follow-up.

^c^ Values are relative risks with 95% CIs.

### Association of Family Member–Identified Palliative Care Needs With Outcomes

In the fully adjusted models (Table 2), family members who had identified at least 1 need at enrollment compared with those who did not had significantly higher depressive symptoms at follow-up (difference in means for PHQ-2, 0.75 points; 95% CI, 0.24 to 1.25 points; *P* = .004). We found no statistically significant association between identified needs and family anxiety or depressive symptoms at enrollment. Identification of at least 1 palliative care need by family members was associated with lower patient QOL (difference in means, −17.08 points; 95% CI, −33.64 to −0.52 points; *P* = .04).

Among individual family-identified needs (Table 3), presence of pain or other distressing symptoms in the patient was significantly associated with higher family anxiety symptoms at follow-up (difference in means for GAD-7, 1.60 points; 95% CI, 0.02 to 3.19 points; *P* = .047). The family-identified need to readdress goals of care was significantly associated with greater goal discordance (RR, 1.59; 95% CI, 1.03 to 2.44; *P* = .04) at enrollment and with lower anxiety at follow-up (difference in means for GAD-7, −3.25 points; 95% CI, −4.62 to −1.88 points; *P* < .001).

## Discussion

In this single-center prospective cohort study of patients with SABI and their family members, palliative care needs in the days following ICU admission were high, and family members more often identified a palliative care need than clinicians. Across all measured domains, agreement on the presence or type of palliative care needs between family members and clinicians was poor. We also found a high proportion of poor outcomes, some of which were associated with identification of a palliative care need early during the ICU stay.

Overall, our findings suggest that clinicians and family members may benefit from improved communication about palliative care needs early on during the ICU stay. For example, more than half of family members were concerned that their loved one was experiencing pain or other distressing symptoms, while this was a need rarely identified by clinicians. Different interpretations of patient’s reactions and experience or different knowledge about how symptoms are managed suggest an important opportunity for clinicians to partner with family members and explore each other’s perspective on patient symptoms and their management. On the other hand, family members rarely identified a need to readdress goals of care, while this was identified by one-quarter of clinician teams. The concept of goals of care may not be as clear to patients and family members.^25^

Asking clinicians to identify needs during morning work rounds did not appear to be associated with poor psychological outcomes, but it was associated with other concerning outcomes, including goal-discordant care and decisional regret. However, we only analyzed the SuPPOrTT checklist completed on the day of enrollment, and needs or awareness of needs may have been different on other days. In addition, exploring these questions among the clinical team on a daily basis may have unmeasured benefits, such as higher quality of communication among clinicians or more documented family meetings, as suggested in a prior study.^18^ Future research should investigate means to quickly identify perceived goal-discordant care throughout the hospitalization, including after leaving the ICU, to address it, and to reduce decisional regret.

Asking family members their perception of needs may help to identify people at increased risk for poor psychological outcomes, including symptoms of anxiety or depression. When family members identified pain or other distressing symptoms in their loved one, these family members were more likely to experience symptoms of anxiety at follow-up compared with those family members who did not identify this need. Only a small proportion of family members identified the need to readdress goals of care, but if they did, they were more likely to rate the patient’s care as goal-discordant and less likely to report anxiety at follow-up. Goal-discordant care in the acute setting is most often due to unwanted life-sustaining treatment,^3^ indicating need for further research into long-term impacts of this perception.

This study suggests that clinicians may not understand the needs of their patients and family members. This understanding could be of critical importance, as it may help identify opportunities for interventions to improve the experience and long-term outcomes of family members.

### Limitations

This study has several important limitations. First, the SuPPOrTT checklist covers multiple domains and may have been interpreted differently by different participants. Furthermore, the degree to which clinicians have knowledge about, and are skilled in, palliative care was unlikely homogeneous across patient-family dyads, although this variation is somewhat mitigated by the team approach to needs assessment during daily work rounds. Second, the associations in this observational study cannot be assumed to represent causation and may be influenced by unmeasured confounders. The time from admission to enrollment may have also affected our conclusions. Third, 1 in 5 participants (22.5%) were lost to follow-up, and missingness was higher for certain outcomes (Tables 2 and 3), which may result in a response bias as family members with psychological distress may be less likely to respond to a survey request or may be less likely to complete questions about difficult topics. Fourth, most patients in this single-center study where non-Hispanic White, which limits generalizability and contributes to the lack of information about risk factors and outcomes in minoritized racial and ethnic individuals. Additionally, in this exploratory study, the significant associations could be due to multiple comparisons. Future studies with larger numbers and greater diversity are needed with the ultimate goal of finding ways to reduce the risk of adverse outcomes in family members of patients with SABI.

## Conclusions

In this study, palliative care needs were common in patients with SABI and their family members, although family members and clinicians did not perceive the same needs. Patient pain or distressing symptoms were commonly identified by family and rarely by clinicians. Family identification of palliative care needs was associated with worse psychological symptoms in family members several months after enrollment. Our findings highlight the need for clinicians to improve their understanding and communication around palliative care needs. Further research is needed to identify interventions that can improve communication about palliative care needs and outcomes of family members.

## Notes

Supplement 1. **eTable 1.** Information Collected and Source of Data Collection at Enrollment and Follow-up **eTable 2.** Associations Between Identified Palliative Care Needs and Outcomes, Unadjusted and Adjusted

Supplement 2.
Data Sharing Statement
