# Supplementary material for: Identification of Palliative Care Needs and Mental Health Outcomes Among Family Members of Patients With Severe Acute Brain Injury
Source: JAMA Netw Open. 2023 Apr 25;6(4):e239949. doi: 10.1001/jamanetworkopen.2023.9949 (PMC10130947; doi:10.1001/jamanetworkopen.2023.9949)
Supplement: Supplement 1. — eTable 1. Information Collected and Source of Data Collection at Enrollment and Follow-up eTable 2. Associations Between Identified Palliative Care Needs and Outcomes, Unadjusted and Adjusted [file jamanetwopen-e239949-s001.pdf]

Supplemental Online Content

Plinke WV, Buchbinder SA, Brumback LC, et al. Identification of palliative care needs and mental health outcomes among family members of patients with severe acute brain injury. *JAMA Netw Open.* 2023;6(4):e239949. doi:10.1001/jamanetworkopen.2023.9949

eTable 1. Information Collected and Source of Data Collection at Enrollment and Follow-up

eTable 2. Associations Between Identified Palliative Care Needs and Outcomes, Unadjusted and Adjusted

This supplemental material has been provided by the authors to give readers additional information about their work.

© 2023 Plinke WV et al. *JAMA Network Open.*

eTable 1. Information Collected and Source of Data Collection at Enrollment and Follow-up
References are listed in the manuscript.

Measures	Collected at		Comments
	Enrollment	Follow up	
**Outcomes**			
Patient Health Questionnaire-2 (PHQ2)[18]	x	x	Scored from 0-6 with a score of 3 or higher suggesting a high level of depressive symptoms
General Anxiety Disorder-7 (GAD7)[19]	x	x	Scored 0-21 with scores 10 or higher suggesting moderate anxiety or worse
Family satisfaction in the ICU (FS-ICU)[20]	x		A validated 24-item tool ranging from 0-100 with higher scores reflecting greater satisfaction
Goal-discordant care from Support study[3,22]	x		Agreement on two questions about desired goals or care and actual goals of care
Decision regret scale (DRS)[21]		x	A 5-item scale with scores range from 0 to 100 and higher scores indicate greater regret
Modified Rankin scale (mRS)[xx]		x	A 7-point disability scale with possible scores ranging from 0 to 6 with 0 no disability and 6 death
Quality of life (Euro-QoL)[xx]		x	Visual analog scale with higher scores on a scale from 0-100 reflecting better QOL
Intensive care unit length of stay		x	Determined by review of electronic health records (EHR)
Death at follow up		x	Determined at follow up with family member and/or from EHR
**Characteristics**			
Self-report by family members	x		Age, gender, race, ethnicity, level of education, relationship to the patient
From medical records for patient	x		Age, diagnosis, race, ethnicity, and disease severity (Glasgow Coma Scale at enrollment and modified Rankin scale at follow up)

© 2023 Plinke WV et al. *JAMA Network Open.*

eTable 2. Associations Between Identified Palliative Care Needs and Outcomes, Unadjusted and Adjusted

	Unadjusted		Adjusted[a]	
	β (95% CI)	p value	β (95% CI)	p value
**Clinician Identification of at least one PC need**				
PHQ2 at Enrollment (n=188)	0.55 (0.00 - 1.10)	0.05	0.42 (-0.20 - 1.04)	0.19
PHQ2 at Follow-Up (n=150)	-0.12 (-0.67 - 0.42)	0.65	-0.40 (-0.93 - 0.14)	0.14
GAD7 at Enrollment (n=188)	1.59 (-0.09 - 3.29)	0.06	1.35 (-0.55 - 3.24)	0.16
GAD7 at Follow-Up (n=147)	-0.03 (-1.59 - 1.54)	0.97	-0.30 (-1.96 - 1.36)	0.72
**Goal Discordance (RR) (n=203)**	**1.48 (1.04 - 2.10)**	**0.03***	**1.74 (1.20 - 2.53)**	**0.004***
FSICU (n=187)	-0.39 (-3.65 - 2.86)	0.81	-0.52 (-4.42 - 3.37)	0.79
ICU LOS (n=209)	1.09 (-2.04 - 4.22)	0.49	1.65 (-1.81 - 5.12)	0.35
mRS at Follow-up (n=198)	0.48 (0.05 - 0.91)	0.03*	0.07 (-0.39 - 0.53)	0.76
Death at follow-up (RR) (n=198)	1.44 (1.05 - 1.98)	0.02*	1.11 (0.80 - 1.53)	0.54
QoL at Follow-Up (n=78)	-5.18 (-15.74 - 5.38)	0.33	-2.72 (-13.80 - 8.36)	0.63
**Dec. Regret at Follow-up (n=144)**	**10.06 (3.21 - 16.91)**	**0.004***	**17.33 (5.42 - 29.25)**	**0.005***
**Clinician Identification of Need for Social/Spiritual Support**				
**PHQ2 at Enrollment (n=188)**	**0.97 (0.25 - 1.69)**	**0.009***	**0.87 (0.10 - 1.63)**	**0.03***
PHQ2 at Follow-Up (n=150)	0.57 (-0.13 - 1.27)	0.11	0.49 (-0.17 - 1.15)	0.14
GAD7 at Enrollment (n=188)	2.27 (0.04 - 4.50)	0.045*	2.13 (-0.18 - 4.46)	0.07
GAD7 at Follow-Up (n=147)	-0.23 (-2.11 - 1.66)	0.81	-0.20 (-2.09 - 1.69)	0.83
Goal Discordance (RR) (n=203)	0.84 (0.55 - 1.27)	0.41	0.98 (0.62 - 1.55)	0.93
Dec. Regret at Follow-up (n=144)	4.25 (-4.96 - 13.45)	0.36	4.84 (-3.38 – 13.07)	0.24
**Clinician Identification of Pain/Distressing Symptom**				
PHQ2 at Enrollment (n=188)	-0.29 (-1.36 - 0.78)	0.60	-0.31 (-1.43 - 0.80)	0.58
PHQ2 at Follow-Up (n=150)	-0.01 (-0.98 - 0.95)	0.97	0.20 (-0.74 – 1.13)	0.68
GAD7 at Enrollment (n=188)	-0.08 (-3.61 - 3.44)	0.96	-0.08 (-3.74 - 3.59)	0.97
**GAD7 at Follow-Up (n=147)**	**3.31 (0.06 - 6.57)**	**0.046***	**3.60 (0.18 - 7.03)**	**0.04***
Goal Discordance (RR) (n=203)	1.35 (0.85 - 2.14)	0.21	1.52 (0.91 - 2.56)	0.11
**Dec. Regret at Follow-up (n=144)**	**18.78 (-1.82- 39.39)**	**0.07**	**22.77 (3.27- 42.28)**	**0.02***
**Clinician Identification of Concern about Prognosis/Treatment Options**				
PHQ2 at Enrollment (n=188)	0.49 (-0.10 - 1.07)	0.1	0.35 (-0.33 - 1.03)	0.31

	Unadjusted		Adjusted[a]	
	β (95% CI)	p value	β (95% CI)	p value
**Family Identification of at least one PC need**				
PHQ2 at Enrollment (n=188)	-0.04 (-1.02 - 0.93)	0.93	0.12 (-0.84 - 1.08)	0.80
**PHQ2 at Follow-Up (n=150)**	**0.91 (0.31 - 1.51)**	**0.003***	**0.75 (0.24 - 1.25)**	**0.004***
GAD7 at Enrollment (n=188)	1.39 (-0.90 - 3.68)	0.23	1.87 (-0.44 - 4.18)	0.11
GAD7 at Follow-Up (n=147)	1.97 (0.05 - 3.88)	0.04*	1.56 (-0.51 - 3.63)	0.14
Goal Discordance (RR) (n=203)	0.87 (0.58 - 1.52)	0.55	0.79 (0.53 - 1.20)	0.27
FSICU (n=187)	-3.48 (-6.48 - -0.48)	0.02*	-2.15 (-5.16 - 0.87)	0.16
ICU LOS (n=209)	-2.05 (-7.00 - 2.89)	0.41	-2.14 (-7.49 - 3.21)	0.43
mRS at Follow-up (n=198)	-0.15 (-0.87 - 0.58)	0.69	-0.17 (-0.85 - 0.50)	0.61
Death at follow-up (RR) (n=198)	0.83 (0.55 - 1.26)	0.39	0.90 (0.59 - 1.36)	0.62
**QoL at Follow-Up (n=78)**	**-16.97 (-32.17 - -1.77)**	**0.03***	**-17.08 (-33.64 - -0.52)**	**0.04***
Dec. Regret at Follow-up (n=144)	4.41 (-4.53 - 13.35)	0.33	-1.62 (-16.99 – 13.74)	0.83
**Family Identification of Need for Social/Spiritual Support**				
PHQ2 at Enrollment (n=188)	0.56 (0.01 - 1.12)	0.047*	0.55 (-0.01 - 1.10)	0.05*
PHQ2 at Follow-Up (n=150)	0.32 (-0.22 - 0.87)	0.25	0.34 (-0.19 – 0.88)	0.21
GAD7 at Enrollment (n=188)	1.33 (-0.38 - 3.05)	0.13	1.20 (-0.48 – 2.89)	0.16
GAD7 at Follow-Up (n=147)	0.61 (-0.95 - 2.18)	0.44	0.82 (-0.73 - 2.36)	0.30
Goal Discordance (RR) (n=203)	0.92 (0.65 - 1.29)	0.62	0.96 (0.68 - 1.34)	0.79
Dec. Regret at Follow-up (n=144)	-0.21 (-7.31 – 6.90)	0.95	-1.24 (-8.18 -5.70)	0.72
**Family Identification of Pain/Distressing Symptom**				
PHQ2 at Enrollment (n=188)	0.21 (-0.36 - 0.77)	0.47	0.32 (-0.23 - 0.87)	0.26
PHQ2 at Follow-Up (n=150)	0.21 (-0.34 - 0.77)	0.45	0.04 (-0.50 - 0.58)	0.89
GAD7 at Enrollment (n=188)	1.08 (-0.63 - 2.79)	0.21	1.32 (-0.44 - 3.08)	0.14
**GAD7 at Follow-Up (n=147)**	**1.89 (0.37 - 3.40)**	**0.01***	**1.60 (0.18 - 3.19)**	**0.047**
Goal Discordance (RR) (n=203)	0.94 (0.67 - 1.32)	0.72	0.85 (0.60 - 1.19)	0.34
Dec. Regret at Follow-up (n=144)	4.83 (-2.17 - 11.84)	0.17	2.97 (-4.08 – 10.01)	0.41
**Family Identification of Concern about Prognosis/Treatment Options**				
PHQ2 at Enrollment (n=188)	-0.41 (-1.00 - 0.17)	0.17	-0.38 (-0.96 - 0.19)	0.19

© 2023 Plinke WV et al. *JAMA Network Open.*

PHQ2 at Follow-Up (n=150)	0.07 (-0.50 - 0.64)	0.8	-0.04 (-0.60 - 0.54)	0.89	PHQ2 at Follow-Up (n=150)	-0.22 (-0.81 - 0.37)	0.47	-0.35 (-0.91 - 0.22)	0.22
GAD7 at Enrollment (n=188)	1.85 (0.04 - 3.66)	0.045*	1.61 (-0.48 - 3.70)	0.13	GAD7 at Enrollment (n=188)	0.24 (-1.52 - 2.00)	0.79	0.33 (-1.40 - 2.07)	0.70
GAD7 at Follow-Up (n=147)	0.38 (-1.26 - 2.02)	0.65	0.39 (-1.40 – 2.18)	0.67	GAD7 at Follow-Up (n=147)	-0.02 (-1.60 - 1.57)	0.98	-0.34 (-1.91 - 1.24)	0.67
Goal Discordance (RR) (n=203)	1.20 (0.86 - 1.68)	0.28	1.29 (0.88 - 1.89)	0.19	Goal Discordance (RR) (n=203)	0.99 (0.70 - 1.39)	0.94	0.95 (0.67-1.33)	0.75
**Dec. Regret at Follow-up (n=144)**	**6.67 (0.74 - 14.08)**	**0.08**	**17.58 (4.14 – 31.03)**	**0.01***	Dec. Regret at Follow-up (n=144)	-6.80 (-15.48 - 1.89)	0.12	-15.81 (-34.32 – 2.69)	0.09
**Clinician Identification of Need to re-address/change GOC**					**Family Identification of need to re-address/change GOC**				
PHQ2 at Enrollment (n=188)	0.56 (-0.14 - 1.25)	0.12	0.39 (-0.34 - 1.12)	0.30	PHQ2 at Enrollment (n=188)	0.41 (-0.73 - 1.56)	0.48	0.51 (-0.66 - 1.68)	0.39
PHQ2 at Follow-Up (n=150)	0.75 (0.03 - 1.48)	0.04*	0.51 (-0.22 - 1.24)	0.17	PHQ2 at Follow-Up (n=150)	-0.19 (-1.53 - 1.15)	0.78	-0.75 (-1.72 – 0.22)	0.13
GAD7 at Enrollment (n=188)	2.12 (-0.08 4.32)	0.06	1.92 (-0.49 - 4.33)	0.12	GAD7 at Enrollment (n=188)	0.31 (-3.45 - 4.07)	0.87	0.74 (-3.19 - 4.67)	0.71
GAD7 at Follow-Up (n=147)	0.11 (-1.99 - 2.20)	0.92	-0.00 (-2.25 – 2.24)	0.998	GAD7 at Follow-Up (n=147)	-1.11 (-5.20 - 2.98)	0.59	**-3.25 (-4.62 - -1.88)**	**<0.001***
Goal Discordance (RR) (n=203)	1.23 (0.86 - 1.76)	0.25	1.36 (0.93 - 1.98)	0.12	**Goal Discordance (RR) (n=203)**	**1.72 (1.11 - 2.67)**	**0.016***	**1.59 (1.03 - 2.44)**	**0.035***
Dec. Regret at Follow-up (n=144)	8.99 (-0.84 - 18.82)	0.07	13.82 (-0.29- 27.94)	0.05	Dec. Regret at Follow-up (n=144)	10.32 (-9.23 - 29.87)	0.3	17.36 (-9.05 – 43.78)	0.20

a Fully adjusted models were adjusted for patient age, diagnosis, race/ethnicity, and disease severity (using either GCS or MRS)

© 2023 Plinke WV et al. *JAMA Network Open.*
