# Supplementary material for: Identification of Palliative Care Needs and Mental Health Outcomes Among Family Members of Patients With Severe Acute Brain Injury
Source: JAMA Netw Open. 2023 Apr 25;6(4):e239949. doi: 10.1001/jamanetworkopen.2023.9949 (PMC10130947; doi:10.1001/jamanetworkopen.2023.9949)
Supplement: Supplement 2. — Data Sharing Statement [file jamanetwopen-e239949-s002.pdf]

Data Sharing Statement

Plinke. Identification of Palliative Care Needs and Mental Health Outcomes Among Family Members of Patients With Severe Acute Brain Injury. *JAMA Netw Open*. Published April 25, 2023. doi:10.1001/jamanetworkopen.2023.9949

Data
Data available: Yes
Data types: Deidentified participant data
How to access data: clairejc@uw.edu
When available: With publication

Supporting Documents
Document types: None

Additional Information
Who can access the data: anyone requesting the data
Types of analyses: for any approved purpose
Mechanisms of data availability: after approval of a proposal
